# Improvement of working memory in older adults with mild cognitive impairment after repetitive transcranial magnetic stimulation – a randomized controlled pilot study

**DOI:** 10.3389/fpsyt.2023.1196478

**Published:** 2023-11-30

**Authors:** Adrianna Senczyszyn, Dorota Szcześniak, Tomasz Wieczorek, Julian Maciaszek, Monika Małecka, Bogna Bogudzińska, Anna Zimny, Karolina Fila-Pawłowska, Joanna Rymaszewska

**Affiliations:** ^1^Department of Psychiatry, Wroclaw Medical University, Wrocław, Poland; ^2^Department of Neurology, Wroclaw Medical University, Wrocław, Poland; ^3^Department of Clinical Neuroscience, Wroclaw University of Science and Technology, Wroclaw, Poland

**Keywords:** mild cognitive impairment, transcranial magnetic stimulation, resting-state functional MRI, computerized cognitive training, cognitive function, dorsolateral prefrontal cortex

## Abstract

Repetitive transcranial magnetic stimulation (rTMS) is a noninvasive technique that could improve cognitive function. It is being developed as a non-pharmacological intervention to alleviate symptoms of cognitive deterioration. We assessed the efficacy of rTMS in improving cognitive functioning among people with Mild Cognitive Impairment (MCI) in a partially-blinded, sham-controlled randomized trial. Out of 91 subjects screened, 31 participants with MCI (mean age 70.73; SD = 4.47), were randomly assigned to one of three groups: (A) Active rTMS; (B) Active rTMS with Computerized Cognitive Training RehaCom; and (C) Sham control. The study evaluated cognitive function using the DemTect, FAS, and CANTAB tests before and after the stimulation. The following treatment protocol was applied: 2000 pulses at 10 Hz, 5-s train duration, and 25-s intervals at 110% of resting MT delivered over the left Dorsolateral Prefrontal Cortex (DLPFC) five times a week for 2 weeks. After 10 sessions of high-frequency rTMS, there was an improvement in overall cognitive function and memory, assessed by the DemTect evaluation, with no serious adverse effects. Analysis of differences in time (after 10 sessions) between studied groups showed statistically significant improvement in DemTect total score (time by group interaction *p* = 0.026) in favor of rTMS+RehaCom. The linear regression of CANTAB Paired Associates Learning revealed significant differences in favor of rTMS+RehaCom in three subtests. Our study shows that 10 sessions of rTMS over the left DLPFC (alone as well as combined with Computerized Cognitive Training) can have a positive impact on cognitive function in people with MCI. Further research should investigate the underlying mechanism and determine the optimal parameters for rTMS, which will be important for its efficacy in clinical settings.

## 1 Introduction

Mild cognitive impairment (MCI) is a term used to describe an early stage of memory loss or other cognitive ability loss in individuals who otherwise maintain independent performance in most activities of daily living. It is perceived as a transitional state between normal aging and dementia. According to Petersen’s MCI classification, it comprises four clinical subtypes: (i) single-domain amnestic MCI; (ii) multiple-domain MCI; (iii) single-domain non-amnestic MCI; and (iv) multiple-domain non-amnestic MCI (1). These subtypes indicate differences in clinical outcomes. Both amnestic MCI (i, ii) are more likely to convert into Alzheimer’s disease (AD), while non-amnestic MCI conditions (iii, iv) are instead more likely to convert into other types of dementia, such as vascular dementia or Lewy body dementia (2, 3).

Most often, patients initially notice a decline in the memory regarding daily activities, recent personal experiences, new information, etc., called everyday memory. Their observations are confirmed by neuropsychological assessment since individuals with MCI typically show impairment in delayed recall tasks, involving encoding and retrieval of information (3). Such cognitive deficits are responsible for a decrease in quality of life (QOL) (4, 5) and make them more susceptible to the occurrence of psychiatric conditions such as depression, irritability, and apathy when compared to older adults without cognitive impairment (6). The relationship between cognitive and neuropsychiatric disorders is a complex phenomenon, as cognitive disorders can be a consequence of confrontation with declining cognitive performance, but they can also precede cognitive disorders as in the case of Parkinson’s disease (7) or result from fear of potential impairment (not necessarily already present). In addition, cognitive impairments can mask other disorders, e.g., a depressive disorder and disappear after appropriate pharmacotherapy (8). Since pharmacological treatment for MCI has exhibited no significant effect on cognitive deterioration symptoms (9) establishing the efficacy of nonpharmacological interventions (e.g., cognitive, physiological, dietary, psychosocial, and noninvasive brain stimulation methods) in slowing the transition from MCI to dementia is playing a leading role in aging research (10).

Transcranial magnetic stimulation (TMS) emerges as a noninvasive electrophysiological method of central nervous system stimulation with the potential to enhance cognitive functioning. During TMS electric current is generated in the therapeutic coil, which subsequently generates a magnetic field responsible for a change of electrical field in the brain cortex. A magnetic pulse delivered by the coil penetrates the skin and skull bone in a non-invasive and generally well-tolerated and safe way. Subsequently, due to numerous connections with many other structures, the stimulus spreads into further regions and functional brain networks (11). Single and paired-pulse protocols are most frequently used for research purposes, i.e., to investigate cortical excitability and reactivity, while repetitive TMS (rTMS) is usually employed in treatment protocols (12). Low-frequency rTMS (≤1 Hz) causes inhibition of cortical excitability, whereas high-frequency rTMS (5-20 Hz) leads to increased excitability (13).

So far, the most commonly used cortical target for the therapeutic application of rTMS in MCI or AD-type dementia was the Dorsolateral prefrontal cortex (DLPFC) (3). The DLPFC is involved in such cognitive functions as everyday memory, working memory (14), and executive function (15). Studies involving functional magnetic resonance imaging (fMRI) have shown that high-frequency rTMS increases cortical excitability of the left and right DLPFC before memory tasks, and these changes are associated with the increased metabolic activity of the right DLPFC (16). Even though rTMS studies on cognitive functions have been conducted for more than 10 years, there are still some controversies regarding its efficacy in improving general cognitive functioning (3), the potential mechanism of the improvement of cognitive performance (17), the level of cognitive deterioration for which rTMS is effective (MCI/AD) (9), and the possibility of enhancing its potential via cognitive training pre/post/during the intervention (18).

A 2022 study by Esposito et al. (10) showed significantly increased semantic fluency (*p* = 0.026) and visuospatial (*p* = 0.014) performances after rTMS in the treated group but not in the sham group. These results are in line with a 2023 literature review on noninvasive brain stimulation in Primary Progressive Aphasia by Papanikolau (19), which points toward the application of rTMS having a positive effect in improving symptoms, such as verb production, action naming, phonemic-verbal fluency, grammatical comprehension, written spelling, and semantic features. On the contrary, the results from a 2023 random-effects meta-analysis by A. Miller et al. (20) demonstrated that rTMS significantly improved global cognitive function relative to control groups (*p* = 0.017), however no significant effects were found for individual cognitive domains. Discrepancies regarding cognitive training are also evident, as some studies report its reinforcing effect on stimulation efficacy (21), while others show that an enhanced synergistic effect does not occur when both interventions are used simultaneously (22).

The purpose of the study was to answer the aforementioned concerns emerging in the evaluation of the effects of rTMS. Firstly, it aims to assess the efficacy of rTMS over the left DLPFC in enhancing general functioning as well as selected cognitive domains of elderly patients. General cognitive functioning, which is a primary outcome of the study, is measured by the DemTect total score. Selected cognitive domains (secondary outcome measures) were assessed by the FAS verbal fluency test and a very sensitive computerized measurement of cognitive function, the Cambridge Neuropsychological Test Automated Battery (CANTAB). Secondly, the study is meant to determine whether the incorporation of Computerized Cognitive Training directly after the rTMS sessions may enhance its efficacy in improving cognitive performance. Based on previous research we can suspect that rTMS can lead to long-lasting after-effects in the brain, and therefore it is thought to be able to induce adaptive structural and functional changes to the brain, called neuroplasticity (23). Because both rTMS and cognitive pieces of training can enhance adaptive plastic mechanisms (24), our focus was to determine if a synergistic positive effect could result from the combination of both approaches, as it was suggested by Birba et al. (21). Results of the study are reported in accordance with “Consolidated Standards for Reporting Trials (CONSORT)” guidelines and recommendations.

## 2 Materials and methods

The study was designed as a partially-blinded sham-controlled randomized trial. Patients as well as raters were blinded regarding the type of treatment (active or sham coil). The participants who performed computerized cognitive training are considered partially-blinded, since they were aware that they were training cognitive functions via RehaCom software. Unblinding was permissible only in case of adverse symptoms that threaten the health of the participant. The person applying the stimulation was unblinded due to technical considerations and was not involved in any rating activities. Two independent data entry personnel entered data separately. Any discrepancies between their entries were resolved by referring back to the source data. This double data entry process allowed to identify and rectify data entry errors effectively. To maintain consistency and facilitate data analysis, we employed a standardized coding system for variables and data categories. Access to the research data was restricted to authorized personnel only. The research protocol was reviewed and approved by the Bioethical Committee at Wroclaw Medical University (KB-400/2018/2506). The trial was registered at ClinicalTrials.gov (NCT05730296).

### 2.1 Participants

The recruitment process was carried out through media advertisements and community settings, between January 2020 and December 2022. Interested patients were scheduled for an appointment with a psychologist who provided them with all the information about the study design and rTMS itself. During the appointment, the psychologist carried out a cognitive examination at T1 (before stimulation) and helped those participants who needed some assistance to fill out a paper form application for the clinical trial, providing their contact details and socio-demographic information. Finally, the patients who completed the application form were contacted and examined by a psychiatrist who assessed their mood and anxiety symptoms and verified the inclusion and exclusion criteria.

The inclusion criteria for the study were: (i) absence of other psychiatric disorders (i.e., depression, anxiety disorders), which may affect cognitive performance (GDS-15, 15-item Geriatric Depression Scale; HAM-A 14, 14-item Hamilton Anxiety Scale); (ii) MCI diagnosis according to Petersen’s criteria such as (a) subjective memory impairment over 1–2 years, (b) objective declined memory performance assessed by Montreal Cognitive Assessment Scale, (MoCA) with score between 19–26, (c) preserved general cognitive function based on the initial interview, (d) minimal impairment in activities of daily living based on the initial interview, (iii) age between 55 and 80 years, (iv) given informed consent to participate in the study and commitment to participate in individual sessions according to the treatment protocol.

The exclusion criteria for the study were divided into two groups: specific TMS contradictions and specific MRI contradictions. The former include (i) a positive history of epileptic seizures or a positive family history of epilepsy, (ii) magnetic or ferromagnetic implants, both electronic (e.g., heart/brain stimulators) as well as mechanical (e.g., bone anastomoses) within the head and neck, (iii) previous stroke or head injury with identified neurological deficits, (iv) increased intracranial pressure or a positive history of increased intracranial pressure, (v) occurrence of significant pathologies in the cerebrum area (tumors, hydrocephalus, strokes). The latter include (i) claustrophobia, and (ii) magnetic or ferromagnetic implants, both electronic (e.g., cardiac/brain stimulators) as well as mechanical (e.g., bone anastomoses) within the head and neck.

The patients who completed the psychological and psychiatric evaluation progressed to receive a structural MRI to exclude contraindications to stimulation. All MR examinations were carried out on a 3 Tesla MR scanner (Ingenia Philips Best Netherlands) equipped with 45 mT/m 200 T/m/s gradients and a 32-channel head coil. All patients underwent brain MRI two times: before TMS (structural imaging followed by resting-state functional MRI) and after TMS sessions (only resting-state functional MRI). Structural imaging was performed to search for brain pathologies that could exclude patients from the study and consisted of standard MR sequences such as axial T2-weighted imaging, 3D FLAIR, DWI, and SWI.

### 2.2 Intervention

Patients included in the study were randomized and assigned into one of three groups using the Sealed Envelope online software application:

- rTMS active group
- rTMS+RehaCom active group
- sham control group

The randomization was stratified by age at baseline. Single pulse stimulation was used to find the motor hotspot, using an electromyography (EMG) signal recorded from the flexor digitorum superficialis (with an electrode located on the index finger). The resting motor threshold (MT) was determined afterward, similarly based on the EMG signal. After MT determination (defined as % of the device output needed to elicit a motor response in ≥50% of the attempts), the stimulation point (target) was set, by moving the coil 6 cm to the front from the determined hotspot. The following treatment protocol was applied (in both active and sham groups): 2000 pulses at 10 Hz, 5-s train duration, and 25-s intervals at 110% of resting MT delivered five times a week for 2 weeks (10 sessions). For the control group, we used a sham coil generating a minimal magnetic field affecting only adjacent tissues (scalp). PowerMag 100 lab device (Mag&More, Munich, Germany) applied in this research, along with active and sham coils of the figure of eight, provided by the same manufacturer. The high frequency (hf) rTMS protocol was ascertained based on previous research (3).

For participants who were allocated to the rTMS+RehaCom group, we employed the software RehaCom (25), which is a modular, interactive program designed to train cognitive abilities. The system includes procedures to train and improve attention, memory, visuospatial processing, and executive functions. The therapist’s interface allows for the introduction and retrieval of personal and clinical information of the patients, the design of individual subprograms, including the individualized level of difficulty, and the collection of data. The training plan was standardized, as each participant performed a different set of exercises each day, programmed in advance by the experimenter for 10 days of stimulation. The training was performed under the supervision of specialists for 30 min just after each TMS session.

### 2.3 Measures

At the stage of the inclusion to the study, symptoms of cognitive decline were diagnosed using the Montreal Cognitive Assessment test and Clinical Dementia Rating Scale. Additionally, symptoms of anxiety and depression, which may negatively affect cognitive functions, were assessed by the 14-item Hamilton Anxiety Scale and the 15-item Geriatric Depression Scale, respectively. Next, the severity of cognitive decline was assessed at two points in time: T1 – before stimulation, and T2 – at the end of stimulation, using the DemTect test for general cognitive functioning (primary outcome) and CANTAB with the Verbal Fluency Test FAS for selected cognitive domains (secondary outcome).

#### 2.3.1 Inclusion measurements

*MoCa (Montreal Cognitive Assessment)* is a screening tool created to identify cognitive impairment. Ziad Nasreddine created this assessment in 1996 as an alternative to the Mini-Mental State Examination. MoCA is a recommended test for MCI detection (26). The cut-off point for MCI is ≤26. MoCA assesses several cognitive domains: orientation, memory, naming, visuospatial functions, vigilance, language, abstract thinking, and alternating trial-making (27).

*CDR (Clinical Dementia Rating Scale)* is a clinical tool for dementia assessment, developed at Washington University School of Medicine. It estimates six cognitive domains: Memory, Orientation judgment, Community Affairs, Home and Hobbies, and Personal Care (28). In addition to the rating for each domain, an overall CDR score may be calculated through the use of the algorithm. In this study, the 0.5 over score was used as a cut-off for MCI.

*GDS-15 (15-Item Geriatric Depression Scale)* is a screening tool used to assess depression symptoms. Of the 15 items, 10 (question numbers 2, 3, 4, 6, 8, 9, 10, 12) indicate the presence of depression symptoms when answered positively, while the rest (1, 5, 7, 11, 13) indicate depression when answered negatively. Scores of 0–4 are considered normal; 5–8 indicate mild depression; 9–11 indicate moderate depression; and 12–15 indicate severe depression. The GDS was found to have a 92% sensitivity and an 89% specificity when evaluated against diagnostic criteria (29). To be included in the study patients needed to score < 7.

*HAM-A 14 (14-Item Hamilton Anxiety Scale)* is a scale widely used by clinicians for patients’ anxiety rates. It was originally published by Max Hamilton in 1959. The scale consists of 14 items, each defined by a series of symptoms, and measures both psychic anxiety (mental agitation and psychological distress) and somatic anxiety (physical ailments related to anxiety) (30). This scale allows us to estimate the extensiveness of anxiety and is still widely used in clinical settings. The cut-off score in the study was <8.

#### 2.3.2 Study outcomes measurements

*DemTect (Demenz-Test)* is a brief (8–10 min), easy-to-administer screening test for dementia comprising five short subtests (10-word list repetition, number transcoding, semantic word fluency task, backward digit span, delayed word list recall) (31). Its transformed total score (maximum 18) is independent of age and education. DemTect allows one to decide whether cognitive performance is age adequate (13–18 points), suggests MCI (9–12 points,) or dementia (8 points or below) (32).

*Verbal Fluency FAS Test* is a measure of phonemic word fluency, which is a type of verbal fluency. Verbal fluency facilitates information retrieval from memory. Successful retrieval requires executive control over such cognitive processes as selective attention, internal response generation, self-monitoring, and self-control. In FAS, by requesting an individual to orally produce as many words that begin with the letters F, A, and S as possible, phonemic fluency is assessed, within a prescribed time, usually 1 min (33).

*CANTAB (the Cambridge Neuropsychological Test Automated Battery)* was created to assess cognitive deficits in patients with neurodegenerative diseases or brain damage (34). Studies show that this tool is a reliable and valid clinical assessment. What is more, its method of administration is exceptionally standardized, which results in fewer variations due to experimenter change or error (35). The Alzheimer battery used in this study estimates cognitive functions in seven domains: Motor Screening Task (MOT): 2 min, Reaction Time (RTI): 3 min, associate learning (PAL): 8 min, Spatial Working Memory (SWM): 4 min, Pattern Recognition Memory (PRM): 4 min, Delayed Matching to Sample (DMS): 7 min and Rapid Visual Information Processing (RVP): 7 min. It takes 35 min to complete the Alzheimer’s battery.

### 2.4 Statistical analysis

The Shapiro–Wilk test and visual assessment were used to analyze the normality of the data. Demographic characteristics at baseline were compared using the Fisher exact test for independent samples (gender, place of residence, education, work, marital status) and the Kruskal-Wallis tests (age, MoCA, HAM-A, GDS scores). Analysis of changes between T1 T2 in FAS, DemTect, and CANTAB was performed using ng Wilcoxon signed-rank test for paired data. Additionally, multivariate mixed models were used to assess differences over time between groups. The level of statistical significance was set at 0.05. Calculations were made using the R for Windows package (version 4.2.2.).

## 3 Results

### 3.1 Consort diagram flow

**Figure 1:**
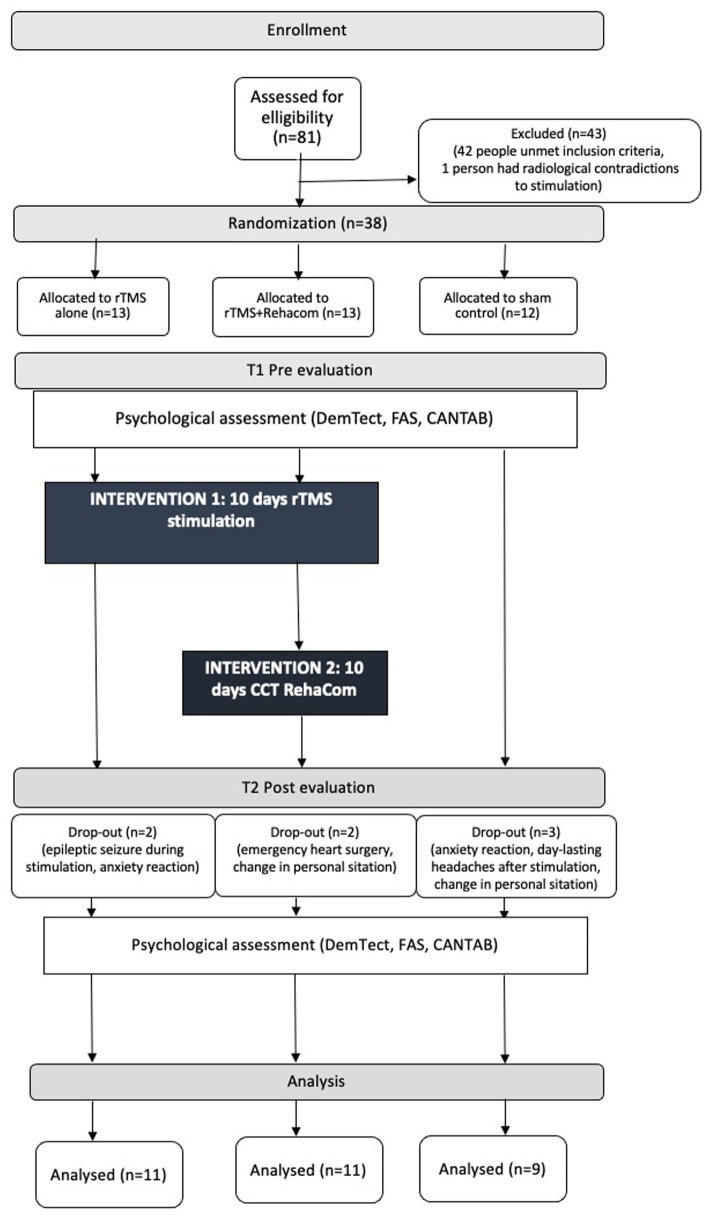
The consort diagram flow of the study.

Out of 81 subjects screened by a psychologist, 42 did not fulfill the enrollment criteria. Among the subjects left, 39 proceeded to get an MRI. One person was excluded from the study at this point due to radiological contradiction, yielding a total number of 43 participants excluded from the study. Next, 38 participants were enrolled in the study and randomly assigned into one of three groups: (A) Active rTMS (*n* = 13); (B) Active rTMS with Computerized Cognitive Training RehaCom (*n* = 13); and (C) Sham control (*n* = 12). Six patients dropped out of the study during the first few sessions and these individuals were excluded due to their inability to follow the procedure protocol. The causes of drop-out were as following: anxiety reaction during stimulation (2 people), Change in personal situation (2 people), 1 day-lasting headache after stimulation (one person). One person resigned before the first rTMS session due to emergency heart surgery, with a total of 1 people who could not participate in the entire stimulation process. In the end a total number of 31 participants finished the protocol. The CONSORT diagram flow of the study design can be found in Figure 1.

### 3.2 Characteristics of the studied group

All of the participants lived in a big city. Above 63% of patients in every group were married. The vast majority had middle or higher education and were retired. More than half of the participants were men. At baseline, groups were homogeneous in terms of global cognitive status and in terms of severity of anxiety and depressive symptoms, failing to meet the criteria for a diagnosis of either disorder. Furthermore, there were no significant differences between the randomized groups regarding the severity of cognitive deterioration at baseline (T1) measured with DemTect, FAS, and CANTAB. Detailed clinical and demographic characteristics as presented in Table 1. Specific data [M (SD)] on CANTAB and, FAS, DemTect scores in T1 and T2 due to their multiplicity are given in Supplementary Table S1.

**Table 1:** Baseline characteristics of the participants.

Characteristics	rTMS	rTMS+RehaCom	Sham	*p*-value
	(*n* = 11)	(*n* = 11)	(*n* = 9)	(*p* < 0.05)
Age (mean, SD)	70.73 (±4.47)	70.64 (±3.14)	71.62 (±5.71)	0.932^a^
Men (*n*, %)	6 (54.5)	7 (63.6)	8 (88.9)	0.308^b^
Education level (*n,* %)				0.122^b^
University	4 (36.4)	4 (36.4)	1 (11.1)	
Secondary	6 (54.5)	7 (63.6)	8 (88.9)	
Elementary	1 (9.1)	0 (0.00)	0 (0.00)	
Marital status (*n*, %)				0.761^b^
Widowed	2 (18.2)	1 (9.1)	0 (0.0)	
Divorced	2 (18.2)	1 (9.1)	1 (11.1)	
Married	7 (63.6)	9 (81.8)	7 (77.8)	
Single	0 (0.0)	0 (0.0)	1 (11.1)	
Town dweller (*n*, %)	11 (100.0)	11 (100.0)	9 (100.0)	>0.999^a^
Retirement (*n*, %)	10 (90.9)	10 (90.9)	8 (88.9)	>0.99^a^
GDS 15 (mean, SD)	4.00 (1.26)	4.00 (1.55)	4.44 (0.73)	0.440^a^
MoCA (mean, SD)	24.82 (1.17)	23.82 (2.71)	24.56 (1.74)	0.561^a^
HAM-A 14 (mean, SD)	5.36 (3.26)	6.00 (2.57)	5.11 (2.98)	0.868^a^
CDR	0.5	0.5	0.5	

Values are given as means (SD) or *n* (%). rTMS: experimental rTMS alone group, rTMS + RehaCom: experimental group with Computerized Cognitive Training RehaCom. sham, sham control group; MoCA, Montreal Assessment Cognitive Scale; DemTect, Demenz Scale; GDS-15, Geriatric Depression Scale; HAM-A 14, Hamilton Anxiety Rating Scale; CDR, Clinical Dementia Rating Scale.

a Kruskal Wallis test.

b Fisher’s test.

### 3.3 Tolerability and safety

rTMS at 10 Hz with 110% of the MT was relatively well tolerated. However, one patient experienced an epileptic seizure during the first session of rTMS, which significantly increased the rate of serious adverse effects in our study to 4.5%. The patient was subsequently counted as drop-outs of the study. 6 patients in the control group and 12 in both experimental groups analyzed together reported some side effects after the intervention, which included headache, insomnia, pain in the area of stimulation, and a burning sensation on the scalp. The number of adverse effects in the experimental and control groups was similar. As stimulation progressed, patients reported fewer adverse effects. For the analysis of TMS side effects, the experimental groups were combined, as we did not expect any somatic side effects caused by computerized training (see Table 2). These side effects did not require medical intervention other than the occasional administration of analgesics.

**Table 2:** Side-effects after rTMS.

Side-effects	Session number	1	*p* value	5	*p* value	10	*p* value
	Group	*n* (%)	(*p* < 0.05)	*n* (%)	(*p* < 0.05)	*n* (%)	(*p* < 0.05)
Seizure	rTMS	1 (4.5)	1^a^	0 (0)	1^a^	0 (0)	1^a^
	Sham	0 (0)		0 (0)		0 (0)	
Insomnia	rTMS	0 (0)	0.29^a^	0 (0)	0.29^a^	0 (0)	1^a^
	Sham	1 (11.1)		1 (11.1)		0 (0)	
Burning scalp	rTMS	4 (18.2)	0.61^a^	2 (9.1)	0.56^a^	1 (4.5)	0.50^a^
	Sham	2 (22.2)		2 (22.2)		1 (11.1)	
Headache	rTMS	3 (13.6)	0.61^a^	0 (0.0)	1^a^	1 (11.1)	1^a^
	Sham	2 (22.2)		0 (0)		0 (0)	
Scalp pain	rTMS	4 (18.2)	1^a^	2 (9.1)	1^a^	1 (4.5)	1^a^
	Sham	1 (11.1)		0 (0)		0 (0)	

Values are given as *n* (%). rTMS, experimental groups; sham, sham control group.

a Fisher’s test.

### 3.4 Linear regression for main outcome variables

A comparison of the efficacy of rTMS alone and rTMS+RehaCom in active and sham stimulation conditions was performed using linear regression with an interaction term. The subtests and total scores of DemTect, FAS, and CANTAB were used as outcome measures.

**Figure 2:**
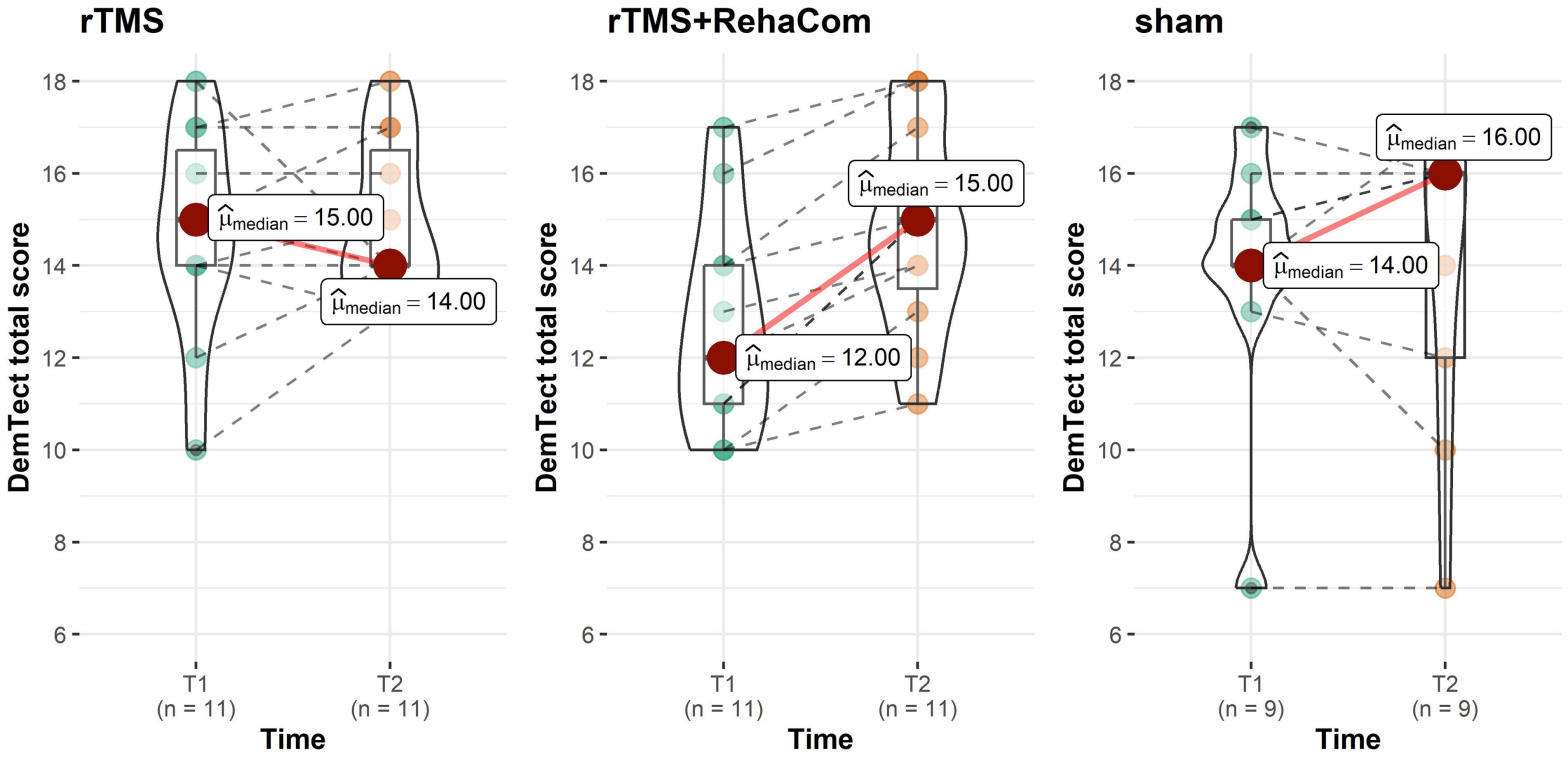
The DemTect total scores (T1 T2).

Analysis of differences in time between studied groups showed evidence for a statistically significant improvement in DemTect total score [time by group interaction *p* = 0.026, T1 mean score (SD) = 11.82 (1.66), T2 mean score (SD) =13.18 (1.72)] in favor of rTMS+RehaCom (Table 3). Moreover, the detailed analysis of individual subtests of the DemTect scale indicates an upward trend towards the significant difference between groups measured over time (T1 vs. T2) in immediate recall DemTect; the sham group performed almost significantly poorer than experimental groups (*p* = 0.068), losing on average 1.34 points in T2, while experimental groups performed better in T2 (rTMS: 1.38 points, RehaCom 1.36 points) (see Figure 2). This trend is most likely responsible for a statistically significant change in the overall DemTect score. There were no statistically significant differences betwee T1, T2 in FAS scores in experimental groups in comparison to a control group (see Table 3 with linear regression model).

**Table 3:** Linear mixed model analysis results (T1, T2).

Tool	Interaction description	Linear mixed model analysis – interaction effect			Effect size
		Beta	95% CI	*p*-value	*η* ^2^
Swms 6	T1	–	–	–	0.001
	T2	−0.591	−1.261, 0079	0.083	
	rTMS	–	–	–	
	rTMS+RehaCom	−0.700	−1.386, −0.014	**0.046**	6.121e-04
	sham	−0.278	−0.982, 0.427	0.432	
	T2 * rTMS +RehaCom	1.346	0.375, 2.318	**0.008**	0.130
	T2 * sham	0.591	−0.394, 1.576	0.234	
Palta 4	T1	–	–	–	0.008
	T2	0.455	−0.472, 1.383	0.322	
	rTMS	–	–	–	
	rTMS+RehaCom	0.08	−0.210, 1.830	0.117	8.756e-04
	sham	0.132	−0.916, 1.181	0.801	
	T2 * rTMS +RehaCom	−1.505	−2.851, −0.160	**0.030**	0.019
	T2 * sham	−0.233	−1.596, 1.129	0.727	
Palte 4	T1	–	–	–	0.044
	T2	1.296	−1.689, 4.281	0.380	
	rTMS	–	–	–	
	rTMS+RehaCom	3.032	−0.16,4 6.229	0.063	0.052
	sham	−0.401	−3.685, 2.883	0.807	
	T2 * rTMS +RehaCom	−4.933	−9.265, −0.601	**0.027**	
	T2 * sham	−1.074	−5.463, 3.316	0.619	0.045
Paltea 4	T1	–	–	–	0.025
	T2	1.296	−1.689, 4.281	0.380	
	rTMS	–	–	–	0.055
	rTMS+RehaCom	3.032	−0.164, 6.229	0.063	
	sham	−0.401	−3.685, 2.883	0.807	
	T2 * rTMS +RehaCom	−4.933	−9.265, −0,601	**0.027**	0.190
	T2 * sham	−1.1	−5.5, 3.3	0.6	
Prmpci	T1	–	–	–	0.012
	T2	−10.379	−19.966, −0.792	**0.034**	
	rTMS	–	–	–	
	rTMS+RehaCom	−10.38	−20.723–1.550	**0.024**	0.134
	sham	2.499	−7.582, 12.580	0.621	
	T2 * rTMS +RehaCom	15.681	2.286, 29.076	**0.023**	0.091
	T2 * sham	8.527	−5.576, 22.629	0.231	
F	T1	–	–	–	0.056
	T2	0.364	−0.184, 0.911	0.184	
	rTMS	–	–	–	
	rTMS+RehaCom	0.455	−2.474, 3.383	0.753	0.028
	sham	−0.646	−3.733, 2.441	0.672	
	T2 * rTMS +RehaCom	0.000	−0.774, 0.774	>0.99	0.061
	T2 * sham	−0.475	−1.290, 0.341	0.243	
A	T1	–	–	–	0.101
	T2	1.091	−0.136, 2.318	0.079	
	rTMS	–	–	–	
	rTMS+RehaCom	−0.27	−3.065, 2.519	0.844	0.056
	sham	−1.2	−4.115, 1.771	0.424	
	T2 * rTMS +RehaCom	−0.27	−2.008, 1.463	0.750	0.053
	T2 * sham	−1.1	−2.920, 0.738	0.232	
S	T1	–	–	–	0.118
	T2	1.455	−0.334, 3.243	0.107	
	rTMS	–	–	–	
	rTMS+RehaCom	0.273	−3.869, 4.414	0.894	0.021
	sham	−1.404	−5.869, 3.000	0.518	
	T2 * rTMS +RehaCom	−1.091	−3.620, 1.439	0.385	0.030
	T2 * sham	−0.232	−2.899, 2.434	0.860	
FAS total	T1	–	–	–	0.204
	T2	2.909	0.538, 5.280	**0.018**	
	rTMS	–	–	–	
	rTMS+RehaCom	0.455	−8.064, 8.973	>0.914	0.038
	sham	−3.222	−12,201, 5.757	0.469	
	T2 * rTMS +RehaCom	−1.364	−4.716, 1.989	0.412	0.042
	T2 * sham	−1,798	−5.332, 1.736	0.306	
Demtect 1	T1	–	–	–	0.010
	T2	1.182	−0.634, 2.998	0.198	
	rTMS	–	–	–	
	rTMS+RehaCom	−0.091	−1.908, 1.725	0.920	0.012
	sham	0.758	−1.157, 2.672	0.431	
	T2 * rTMS +RehaCom	0.182	−2.386, 2.750	0.888	0.079
	T2 * sham	−2.515	−5.222, 0.192	0.068	
Demtect 2	T1	–	–	–	0.065
	T2	0.000	−0.149, 0.149	>0.999	
	rTMS	–	–	–	
	rTMS+RehaCom	−0.182	−0.433, 0.069	0.151	0.111
	sham	−0.222	−0.487, 0.042	0.097	
	T2 * rTMS +RehaCom	0.182	−0.229, 0.393	0.089	0.125
	T2 * sham	0.00	−0.223, 0.223	>0.999	
Demtect 3	T1	–	–	–	0.020
	T2	0.182	−2,938, 3.302	>0.906	
	rTMS	–	–	–	
	rTMS+RehaCom	−0.636	−4.470, 3.197	0.740	0.072
	sham	−1.141	−5.183, 2.900	0.573	
	T2 * rTMS +RehaCom	0.091	−4.505, 4.321	>0.967	0.052
	T2 * sham	−2.515	−7.166, 2.136	0.277	
Demtect 4	T1	–	–	–	0.010
	T2	0.00	−0.352, 0.352	>0.999	
	rTMS	–	–	–	
	rTMS+RehaCom	−0.55	−1.227, 0.136	0.113	0.130
	sham	−0.61	−1.324, 0.112	0.096	
	T2 * rTMS +RehaCom	0.27	−0.224, 0.770	0.271	0.081
	T2 * sham	−0.11	−0.635, 0.413	0.411	
Demtect 5	T1	–	–	–	0.125
	T2	0.545	−0.612, 1.703	0.343	
	rTMS	–	–	–	
	rTMS+RehaCom	−0.636	−2.254, 1.081	0.459	0.016
	sham	−0.283	−2.093, 1.528	0.754	
	T2 * rTMS +RehaCom	0.273	−1.364, 1.910	0.735	0.004
	T2 * sham	0.121	−1.604, 1.847	0.887	
Demtect total	T1	–	–	–	0.161
	T2	0.273	−0.791, 1.337	0.604	
	rTMS	–	–	–	
	rTMS+RehaCom	−2.000	−4.160, 0.160	0.069	0.052
	sham	−0.838	−3.115, 1.438	0.460	
	T2 * rTMS +RehaCom	1.727	0.223, 3.223	**0.026**	0.241
	T2 * sham	−0.38	−1.970, 1.202	0.624	

Values are given as Beta coefficient. Bolding indicates a statistically significant score (*p* ≤ 0.05). rTMS, experimental rTMS alone group; rTMS + RehaCom, an experimental group with Computerized Cognitive Training RehaCom; sham, sham control group; FAS, Verbal Fluency Test FAS; DemTect, Demenz Test; Swms6, Palta 4, Palte 4, Paltea 4, Prmpci – subtest from the Cambridge Neuropsychological Test Automated Battery in which Linear Mixed Model Analysis with Interaction Effect was statistically significant.

The linear regression of CANTAB Paired Associates Learning, which assesses visual memory and new learning, revealed significant differences in favor of rTMS+RehaCom in three subtests. In *palta 4*, which measures the total number of attempts made (but not necessarily completed) by the subject during the assessment containing a total of 4 shapes to recall, and in *palte 4* and *paltea 4*, both of which count the total number of attempts made (but not necessarily completed) by the subject during the assessment of a total of 4 shapes to recall [*palta 4* time by group interaction *p* = 0.030, T1 mean score (SD) = 2.90 (1.20), T2 mean score (SD) = 1.89 (0.93), *palte 4* and *paltea 4* time by group interaction *p* = 0.027, T1 mean score (SD) = 6.10 (4.15), T2 mean score (SD) = 2.56 (3.47)]. In each subtest, rTMS+RehaCom group obtained lower scores in T2. Also in Pattern Recognition Memory, which is a test of visual pattern recognition memory in a 2-choice forced discrimination paradigm, statistical analysis showed a significant difference in favor of rTMS+RehaCom *prmpci subtest*, evaluating the number of correct patterns selected by the subject in the immediate forced-choice condition [*prmpci* time by group interaction *p* = 0.023, T1 mean score (SD) = 78.03 (11.35), T2 mean score (SD) = 83.33 (12.91)]. The linear regression on CANTAB Spatial Working Memory, assessing retention and manipulation of visuospatial information, showed a significant difference in favor of the rTMS alone group in the *swms 6* strategic thinking subtest [time by group interaction *p* = 0.008, T1 mean score (SD) = 3.80 (0.92), T2 mean score (SD) = 4.56 (0.73)]. The linear regression performed for other subtests from the CANTAB battery yielded no significant differences in the studied groups between T1 and T2. Due to the amount of data, this information is not included in Table 3.

## 4 Discussion

This study investigated the effects of rTMS (alone as well as combined with Computerized Cognitive Training) over the left DLPFC on cognitive functions in MCI individuals. Cognitive performance at T1 and T2 was evaluated by paper-based (DemTect, FAS) and computer-based (CANTAB) tools. Our study indicates that the administration of 10 sessions of rTMS along with computer-based cognitive training has the potential for significant cognitive improvement among MCI participants that was observed in DemTect total score and several CANTAB subtests in a partially-blind, randomized sham-controlled study. Results from the CANTAB showed that participants received higher scores in T2 in subtests assessing visual memory, new learning, and visual pattern recognition, most associated with working memory. For the other examined cognitive functions (verbal fluency, delayed memory, reaction time) no statistically significant improvements after the rTMS sessions were found. Based on these results the conclusion can be drawn, that 10 sessions of 10 Hz rTMS at 110% MT followed by cognitive training improve working memory.

The recent data of ASL perfusion and resting-state functional magnetic resonance imaging (rs-fMRI) showed that patients with cognitive impairments including MCI show abnormalities in regional cerebral blood flow, which were mainly located in the left posterior cingulate cortex (PCC), the left and right dorsolateral prefrontal cortex (DLPFC), the left inferior parietal lobule (IPL), the right middle temporal gyrus (MTG), the left middle occipital gyrus (MOG), and the left precuneus (PCu) (36). The fact that in our study patients improved mainly in terms of working memory, while almost no significant changes in long-term memory and other cognitive variables were observed, can be interpreted in the light of the previous studies regarding changes in the activity of neural networks after TMS stimulation within the DPLFC. The DLPFC together with the lateral posterior parietal cortex (lPPC) and the Central Executive Network (CEN), regulates executive functions such as working memory and cognitive flexibility (37). Previous studies have shown that TMS within the DLPFC increases activity within CEN and decreases activity within the oppositely correlated Default Mode Network (DMN). The DMN is primarily composed of the dorsal medial prefrontal cortex (mPFC), PCC/PCu, and angular gyrus and is responsible for slow-flowing thoughts, which may explain the improvement in working memory obtained in our study (38).

Another way of describing executive cognitive functions is the Executive Control Network (ECN) consisting of: the DLPFC related to working memory and attention; the inferior parietal lobule (IPL) related to bottom-up attention and episodic memory; the middle frontal gyrus (MFG) related to executive ability; and the middle temporal gyrus (MTG) related to language function (32). The findings imply the effect of rTMS applied to the left DLPFC may have both direct and indirect effects on brain regions activating the working memory-associated network such as connections to the prefrontal and limbic systems. Research by Xiao et al. showed that iTBS applied over the left DPLFC significantly enhanced the brain function connection between the left DLPFC and the left IPL within Alzheimer’s Disease patients, compared to healthy controls (39). In conclusion, previous literature provides vital data indicating the beneficial effect of TMS within the DLPFC on neural networks related to working memory and attention, which contrasts with the relative lack of reports on the positive effect of TMS on other cognitive functions – which was also demonstrated in our study regarding MCI patients.

Interestingly, the enhancement of working memory was noticed only in the rTMS group that also received computer-based cognitive training, which may suggest that rTMS enhances cognitive performance as long as it is combined with an extra procognitive intervention. The obtained results can be interpreted in two ways: either rTMS has an additive effect on the efficacy of cognitive training, or it is the computerized cognitive training itself that led to improved performance, not rTMS. The former standpoint is supported by the fact that both methods change brain activity, so we can suspect that when combined, they may lead to a greater degree of enhancement of cognitive function. Behind this hypothesis is the fact that rTMS is increasingly treated as an adjunctive method for treating mental, emotional, and behavioral disorders, which contributes to strengthening the effect of the first-line therapies (40–43).

In favor of the second position is the fact that cognitive training, having its level tailored to the needs and capabilities of the trainee and taking into account tasks related to the daily life of the subjects, contributes to the improvement of the cognitive performance of MCI patients, as reported by some systematic reviews (44, 45). Computerized Cognitive Training RehaCom applied in the present study is a comprehensive system of software that allows training specific aspects of attention, concentration, memory, perception, and activities of daily living. Its most notable advantages are that, first, it has high ecological validity (its tasks resemble the challenges we face in our daily lives increasing the possibility of transfer of training to contexts beyond the trained tasks), and second, the system is auto-adaptive (the level of these tasks is appropriate to the baseline and to the progress, the patient is making). Therefore, the improvement in working memory might be a result of the implementation of well-designed cognitive training, rather than just rTMS. Cognitive training is also associated with well-documented changes in brain activity in the frontal and parietal cortex and basal ganglia, as well as changes in dopamine receptor density (46). RehaCom training may have potentiated the effect of the rTMS in this study.

Finally, it is worth noting that the study did not assess mood before and after stimulation (but it did measure the presence of depressive symptoms, to exclude subjects whose cognitive impairment may have resulted from depressive disorders). Since the left DLFPC is also stimulated in depression treatment protocols using rTMS (18, 38, 47) we cannot rule out that to some extent the improvement in working memory performance may have been related to the mood improvement.

### 4.1 Limitations of the study

Our study has several limitations. First of all, it involved a relatively small group of participants (n = 31), divided into three study groups, which poses a significant limitation on the generalizability of the results and impacts the conclusions as well as the power of the performed statistical analysis. Secondly, it lacks a fourth study group, participants undergoing cognitive training alone, making it impossible to clearly answer the question of whether computerized training or rTMS is responsible for the improvement in participants’ cognitive performance. Thirdly, the study was partially blinded, since participants from rTMS+RehaCom group were aware that they are training cognitive functions via RehaCom software. Fourthly, despite the exclusion of all but the amnestic MCI subtype, the preliminary analysis of collected neuroimaging data (MRI) suggested the group remained heterogeneous in terms of neuronal deficits. These finding doubt on the possibility of establishing one common protocol for MCI patients, without previous extensive and expensive MRI testing, even when accounting for the MCI subtype. Finally, the study did not include a mood assessment after the intervention. Knowing that the same rTMS protocols over left DLPFC lead to positive clinical outcomes in patients with depression, we cannot eliminate the possibility that to a small extent (those with depressive symptoms were excluded from the study) the improvement in working memory performance might be an indirect consequence of improved affect, the assessment of depressive symptoms at the time of enrollment in the study ensures that participants without moderate to severe depressive symptoms participated in the study.

### 4.2 Implications of research

Based on the results of this study, some implications for future research can be pointed out. There is a need for more studies comparing the rTMS effect with and without additional cognitive stimulation (like memory training). Furthermore, studies comparing the sole effect of cognitive stimulation to combined treatment with rTMS are needed to establish significant clinical implications. Finally, larger studies comparing directly different TMS protocols in MCI treatment are required to determine the most efficient modality of TMS (also including modern variants like theta-burst stimulation or deep TMS). It would be valuable to focus on the effect on various cognitive functions, not exclusively on the memory modality. In addition, future research should focus on the analysis of the mechanism of action of TMS in participants with cognitive impairments, including cognitive reserve and brain network changes.

## 5 Conclusion

This study provides evidence that 10 rTMS sessions combined with individualized computerized cognitive training improve working memory in MCI patients. The area targeted in the study was DLPFC. The exact mechanism of action of rTMS remains unknown, but a prevalent theory involves the induction of long-term potentiation such as plasticity. Enhanced plasticity may make the brain more receptive to cognitive training. These results support the development of noninvasive interventions for persons at risk of dementia, especially since causal treatment is not available to date.

## Data availability statement

The original contributions presented in the study are included in the article/Supplementary material, further inquiries can be directed to the corresponding author.

## Author contributions

AS, DS, and JR were involved in the conceptualization of the study and study design. AS, DS, and JM conducted the data analyses. AS, DS, TW, JM, MM, BB, AZ, KF-P, and JR collaborated on the interpretation of findings and placement in context. The manuscript was drafted by AS. TW, KF-P, AZ, MM, and BB were responsible for editing and refining of the manuscript’s content. All authors contributed to the article and approved the submitted version.
