# Supplementary material for: Improvement of working memory in older adults with mild cognitive impairment after repetitive transcranial magnetic stimulation – a randomized controlled pilot study
Source: Front Psychiatry. 2023 Nov 30;14:1196478. doi: 10.3389/fpsyt.2023.1196478 (PMC10726746; doi:10.3389/fpsyt.2023.1196478)
Supplement: Supplementary file 1 [file Table_1.docx]

| Characteristic | T1 | | | | | T2 | | | | |
| --- | --- | --- | --- | --- | --- | --- | --- | --- | --- | --- |
|  | **N** | **rTMS**  **mean, (SD)** | **rTMS +RehaCom**  **(mean, SD)** | **sham**  **(mean, SD)** | **p-value***^1^* | **N** | **rTMS, N = 11** | **rTMS +RehaCom, N = 11** | **sham, N = 9** | **p-value***^1^* |
| CANTAB subtests: |  | | | | | | | |  |  |
| **palfams** | 29 | 7.00,  (4.57) | 6.00,  (3.74) | 8.44,  (3.84) | 0.44 | 29 | 6.64, (3.11) | 6.67, (5.96) | 8.33, (4.36) | 0.53 |
| **palmets** | 29 | 2.10,  (1.52) | 3.10,  (1.66) | 2.33,  (1.22) | 0.29 | 29 | 2.18, (1.99) | 2.22, (1.30) | 2.22, (1.20) | >0.99 |
| **palnpr** | 29 | 6.80,  (1.69) | 6.20,  (1.75) | 7.56,  (0.88) | 0.19 | 29 | 6.55, (1.57) | 6.44, (1.33) | 7.11, (1.45) | 0.49 |
| **palta** | 29 | 8.40,  (1.90) | 7.80,  (1.48) | 9.44,  (1.81) | 0.15 | 29 | 8.18, (1.94) | 7.44, (0.53) | 8.56, (0.88) | **0.045** |
| **palta2** | 29 | 1.40,  (0.70) | 1.50,  (0.85) | 1.56,  (0.73) | 0.84 | 29 | 1.27, (0.47) | 1.56, (0.73) | 1.33, (0.71) | 0.58 |
| **palta4** | 29 | 2.10,  (1.29) | 2.90,  (1.20) | 2.22,  (0.97) | 0.25 | 29 | 2.55, (1.44) | 1.89, (0.93) | 2.44, (0.73) | 0.38 |
| **palta6** | 29 | 2.50,  (1.58) | 2.00,  (1.83) | 2.78,  (1.09) | 0.64 | 29 | 2.73, (1.62) | 2.89, (1.45) | 2.33, (1.32) | 0.58 |
| **palta8** | 29 | 2.40,  (2.07) | 1.40,  (1.84) | 2.89,  (1.76) | 0.15 | 29 | 1.64, (1.96) | 1.11, (1.69) | 2.44, (1.94) | 0.26 |
| **palte** | 29 | 19.10,  (6.12) | 16.30,  (3.97) | 17.56,  (3.91) | 0.42 | 29 | 18.82, (8.66) | 15.33, (6.96) | 16.22, (4.87) | 0.69 |
| **palte2** | 29 | 0.80,  (1.40) | 0.80,  (1.40) | 0.67,  (0.87) | 0.96 | 29 | 0.45, (0.82) | 1.11, (1.45) | 0.56, (1.33) | 0.48 |
| **palte4** | 29 | 3.10,  (4.28) | 6.10,  (4.15) | 2.67,  (1.94) | 0.12 | 29 | 4.36, (4.39) | 2.56, (3.47) | 2.89, (1.62) | 0.64 |
| **palte6** | 29 | 5.20,  (4.24) | 5.20,  (6.21) | 5.00,  (4.27) | 0.91 | 29 | 7.91, (6.88) | 8.44, (8.00) | 4.22, (5.14) | 0.41 |
| **palte8** | 29 | 10.00,  (9.08) | 4.20,  (5.55) | 9.22,  (6.50) | 0.15 | 29 | 6.09, (8.28) | 3.22, (5.36) | 8.56, (7.45) | 0.28 |
| **paltea** | 29 | 34.30, (16.40) | 39.10, (19.41) | 23.78,  (13.19) | 0.15 | 29 | 37.73, (17.74) | 36.22, (20.44) | 27.78, (17.05) | 0.57 |
| **paltea2** | 29 | 0.80,  (1.40) | 0.80,  (1.40) | 0.67,  (0.87) | 0.96 | 29 | 0.45, (0.82) | 1.11, (1.45) | 0.56, (1.33) | 0.48 |
| **paltea4** | 29 | 3.10,  (4.28) | 6.10,  (4.15) | 2.67,  (1.94) | 0.12 | 29 | 4.36, (4.39) | 2.56, (3.47) | 2.89, (1.62) | 0.64 |
| **paltea6** | 29 | 9.20,  (6.55) | 11.20,  (7.91) | 5.00,  (4.27) | 0.19 | 29 | 11.55, (7.03) | 10.67, (8.14) | 6.44, (7.06) | 0.24 |
| **paltea8** | 29 | 21.20,  (6.53) | 21.00,  (9.12) | 15.44,  (8.09) | 0.24 | 29 | 21.36, (8.81) | 21.89, (9.45) | 17.89, (8.48) | 0.57 |
| **prmclsdd** | 25 | 962.52, (636.91) | 660.27, (283.21) | 1,334.29, (1,017.28) | 0.14 | 26 | 940.50, (742.72) | 760.58, (245.28) | 1,415.16, (1,306.00) | 0.53 |
| **prmclsdi** | 30 | 644.48, (398.57) | 1,080.34, (996.40) | 895.03, (985.05) | 0.47 | 31 | 845.96, (600.95) | 946.49, (713.35) | 743.36, (536.53) | 0.74 |
| **prmmcld** | 25 | 2,459.44, (571.01) | 2,485.10, (586.96) | 2,953.56, (1,411.09) | 0.72 | 26 | 2,488.01, (789.05) | 2,599.11, (375.86) | 2,739.70, (1,055.33) | 0.68 |
| **prmmcli** | 30 | 2,190.77, (482.52) | 2,484.98, (883.58) | 2,293.46, (875.45) | 0.60 | 31 | 2,106.13, (475.74) | 2,413.04, (828.05) | 2,405.54, (1,288.02) | 0.68 |
| **prmmdcld** | 25 | 2,247.94, (492.94) | 2,477.79, (534.35) | 2,872.44, (1,651.72) | 0.57 | 26 | 2,173.95, (600.83) | 2,500.29, (405.64) | 2,318.61, (874.83) | 0.18 |
| **prmmdcli** | 30 | 2,024.75, (417.30) | 2,071.64, (540.13) | 2,026.22, (580.31) | 0.96 | 31 | 1,853.68, (380.75) | 2,108.23, (452.82) | 2,330.83, (1,456.81) | 0.31 |
| **prmpcd** | 25 | 76.85, (10.02) | 58.33, (23.57) | 75.93, (12.11) | 0.11 | 26 | 74.17, (13.29) | 58.33, (19.24) | 78.70, (13.25) | 0.084 |
| **prmpci** | 30 | 89.17, (6.86) | 78.03, (11.35) | 91.67, (9.32) | 0.016 | 31 | 78.79 (14.61) | 83.33 (12.91) | 89.81 (6.95) | 0.17 |
| **rtifesi** | 30 | 0.20,  (0.42) | 0.82,  (2.09) | 0.44,  (0.73) | 0.76 | 30 | 0.82, (1.40) | 0.90, (2.18) | 0.22, (0.67) | 0.46 |
| **rtifesnr** | 30 | 0.00,  (0.00) | 0.00,  (0.00) | 0.00,  (0.00) | - | 30 | 0.00, (0.00) | 0.00, (0.00) | 0.00, (0.00) | - |
| **rtifespr** | 30 | 0.60,  (0.97) | 0.09,  (0.30) | 0.22,  (0.44) | 0.23 | 30 | 0.18, (0.60) | 0.10, (0.32) | 0.11, (0.33) | >0.99 |
| **rtifmdmt** | 30 | 277.80, (69.72) | 363.68, (83.38) | 360.22, (113.62) | 0.082 | 30 | 270.64, (81.03) | 387.75, (50.46) | 334.33, (112.61) | **0.004** |
| **rtifmdrt** | 30 | 397.55, (62.03) | 429.05, (42.85) | 399.00, (52.97) | 0.12 | 30 | 387.55, (50.17) | 423.65, (30.54) | 393.78, (56.13) | 0.078 |
| **rtifmmt** | 30 | 277.10, (63.68) | 371.45, (87.43) | 360.66, (112.63) | **0.050** | 30 | 271.19, (74.34) | 394.84, (57.15) | 338.51, (114.07) | **0.004** |
| **rtifmrt** | 30 | 412.87, (67.32) | 447.28, (59.03) | 415.19, (55.61) | 0.17 | 30 | 406.38, (45.30) | 446.60, (45.81) | 411.46, (59.34) | 0.080 |
| **rtifmtsd** | 30 | 41.94, (17.04) | 58.23, (33.02) | 46.58, (18.57) | 0.45 | 30 | 41.69, (13.18) | 62.36, (28.35) | 51.50, (24.99) | 0.14 |
| **rtifrtsd** | 30 | 80.48, (41.05) | 85.53, (64.49) | 78.15,  (29.99) | 0.96 | 30 | 75.46, (18.97) | 91.92, (68.37) | 73.20, (37.48) | 0.55 |
| **rtiftes** | 30 | 1.20,  (1.81) | 1.09,  (2.12) | 0.78,  (1.39) | 0.82 | 30 | 1.00, (1.79) | 1.00, (2.16) | 0.44, (1.33) | 0.40 |
| **swmbe** | 29 | 23.60,  (6.96) | 17.60,  (7.71) | 24.22,  (3.99) | 0.092 | 29 | 22.00,  (5.48) | 20.67,  (4.03) | 23.33,  (4.36) | 0.53 |
| **swmbe4** | 29 | 2.00,  (1.33) | 1.10,  (1.29) | 2.44,  (0.88) | 0.090 | 29 | 1.91,  (1.30) | 1.33,  (1.50) | 2.11,  (1.27) | 0.46 |
| **swmbe6** | 29 | 6.50,  (2.64) | 4.60,  (3.72) | 8.44,  (2.88) | 0.14 | 29 | 5.36,  (2.84) | 6.78,  (2.44) | 7.78,  (3.67) | 0.19 |
| **swmbe8** | 29 | 15.10,  (4.68) | 11.90,  (4.43) | 13.33,  (2.29) | 0.37 | 29 | 14.73,  (2.76) | 12.67,  (1.66) | 13.44,  (2.07) | 0.27 |
| **swmde** | 29 | 0.60,  (1.58) | 0.50,  (0.85) | 0.89,  (1.45) | 0.78 | 29 | 0.73,  (0.90) | 0.22,  (0.44) | 1.11,  (1.36) | 0.22 |
| **swmde4** | 29 | 0.00,  (0.00) | 0.00,  (0.00) | 0.00,  (0.00) | - | 29 | 0.00,  (0.00) | 0.00,  (0.00) | 0.00,  (0.00) | - |
| **swmde6** | 29 | 0.00,  (0.00) | 0.10,  (0.32) | 0.11,  (0.33) | 0.58 | 29 | 0.09,  (0.30) | 0.56,  (1.33) | 0.22,  (0.44) | 0.65 |
| **swmde8** | 29 | 0.60,  (1.58) | 0.40,  (0.84) | 0.78,  (1.39) | 0.77 | 29 | 0.64,  (0.81) | 0.56,  (1.33) | 0.89,  (1.45) | 0.70 |
| **swmpr** | 29 | 5.00,  (0.00) | 5.00,  (0.00) | 5.00,  (0.00) | - | 29 | 5.00,  (0.00) | 5.00,  (0.00) | 5.00,  (0.00) | - |
| **swms** | 29 | 9.50,  (1.08) | 9.10,  (1.45) | 10.11,  (0.60) | 0.19 | 29 | 9.36,  (1.50) | 10.11,  (1.17) | 10.00,  (1.12) | 0.53 |
| **swms6** | 29 | 4.50,  (0.85) | 3.80,  (0.92) | 4.22,  (0.67) | 0.23 | 29 | 3.91,  (0.70) | 4.56,  (0.73) | 4.22,  (0.67) | 0.12 |
| **swmte** | 29 | 23.80,  (7.22) | 17.80,  (7.55) | 24.56,  (4.45) | 0.089 | 29 | 22.55,  (5.80) | 20.89,  (3.95) | 23.89,  (4.73) | 0.44 |
| **swmte4** | 29 | 2.10,  (1.37) | 1.30,  (1.49) | 2.44,  (0.88) | 0.27 | 29 | 2.00,  (1.48) | 1.33,  (1.50) | 2.11,  (1.27) | 0.45 |
| **swmte6** | 29 | 6.50,  (2.64) | 4.60,  (3.72) | 8.44,  (2.88) | 0.14 | 29 | 5.55,  (2.91) | 6.89,  (2.62) | 7.89,  (3.76) | 0.21 |
| **swmte8** | 29 | 15.20,  (4.83) | 11.90,  (4.43) | 13.67,  (2.45) | 0.36 | 29 | 15.00,  (3.07) | 12.78,  (1.39) | 13.89,  (2.26) | 0.20 |
| **swmwe** | 29 | 0.80,  (1.87) | 0.70,  (0.82) | 1.22,  (2.33) | 0.75 | 29 | 1.27,  (1.19) | 0.44,  (0.73) | 1.67,  (2.29) | 0.24 |
| **swmwe4** | 29 | 0.10,  (0.32) | 0.20,  (0.42) | 0.00,  (0.00) | 0.37 | 29 | 0.09,  (0.30) | 0.00,  (0.00) | 0.00,  (0.00) | 0.44 |
| **swmwe6** | 29 | 0.00,  (0.00) | 0.10,  (0.32) | 0.11,  (0.33) | 0.58 | 29 | 0.27,  (0.47) | 0.22,  (0.67) | 0.33,  (0.71) | 0.78 |
| **swmwe8** | 29 | 0.70,  (1.57) | 0.40,  (0.84) | 1.11,  (2.32) | 0.81 | 29 | 0.91,  (1.04) | 0.22,  (0.44) | 1.33,  (2.40) | 0.31 |
| **dem_tect 1** | 31 | 11.91 (2.17) | 11.82,  (1.66) | 12.67, (1.94) | 0.58 | 31 | 13.09, (3.05) |  |  |  |
| **dem_tect 2** | 31 | 4.00, (0.00) | 3.82,  (0.40) | 3.78, (0.44) | 31 | 31 | 4.00, (0.00) |  |  |  |
| **dem_tect 3** | 31 | 22.36 (5.52) | 21.73,  (4.00) | 21.22, (5.80) | 0.62 | 31 | 22.55, (3.33) |  |  |  |
| **dem_tect 4** | 31 | 4.27, (0.65) | 3.73,  (0.65) | 3.67, (1.12) | 0.18 | 31 | 4.27, (0.65) |  |  |  |
| **dem_tect 5** | 31 | 4.73, (1.85) | 4.09,  (2.51) | 4.44, (1.74) | 0.74 | 31 | 5.27, (1.56) |  |  |  |
| **dem_tect**  **total score** | **31** | **14.73 (2.33)** | **12.73,**  **(2.33)** | **13.89, (2.85)** | **0.11** | **31** | **15.00, (1.73)** |  |  |  |
| **F** | 31 | 10.09, (2.51) | 10.55,  (4.48) | 9.44, (2.65) | 0.84 | 31 | 10.45, (2.30) |  |  |  |
| **A** | 31 | 10.73, (3.23) | 10.45,  (3.42) | 9.56, (2.74) | 0.79 | 31 | 11.82, (2.89) |  |  |  |
| **S** | 31 | 13.18, (3.31) | 13.45,  (5.16) | 11.78, (3.96) | 0.76 | 31 | 14.64, (3.29) |  |  |  |
| **FAS total score** | **31** | **34.00, (7.28)** | **34.45,**  **(11.72)** | **30.78, (8.44)** | **0.79** | **31** | **36.91, (6.27)** |  |  |  |

Values are given as means (SD). rTMS: experimental rTMS alone group, rTMS+RehaCom: experimental group with Computerized Cognitive Training RehaCom, sham: sham control group, MoCA: Montreal Assessment Cognitive Scale, DemTect: Demenz Scale, Palfams, palmets, palnpr, palta, palta2, palta4, palta6, palta8, palte, palte2, palte4, palte6, palte8, paltea, paltea2paltea4, paltea6, paltea8, prmclsdd, prmclsdi, prmmcld, prmmcli, prmmdcld, prmmdcli, prmpcd, prmpci, rtifesi, rtifesnr

rtifespr, rtifmdmt, rtifmdrt, rtifmmt, rtifmrt, rtifmtsd, rtifrtsd, rtiftes, swmbe, swmbe4, swmbe6, swmbe8, swmde, swmde4, swmde6, swmde8, swmpr, swms, swms6, swmte, swmte4, swmte6, swmte8, swmwe, swmwe4, swmwe6, swmwe8 are subtests from CANTAB battery, ^1^Kruskal Wallis test
